# A Novel Hybrid Sequence-Based Model for Identifying Anticancer Peptides

**DOI:** 10.3390/genes9030158

**Published:** 2018-03-13

**Authors:** Lei Xu, Guangmin Liang, Longjie Wang, Changrui Liao

**Affiliations:** 1 School of Electronic and Communication Engineering, Shenzhen Polytechnic, Shenzhen 518060, China; csleixu@szpt.edu.cn (L.X.); wanglongjie@szpt.edu.cn (L.W.); 2 Key Laboratory of Optoelectronic Devices and Systems of Ministry of Education and Guangdong Province, College of Optoelectronic Engineering, Shenzhen University, Shenzhen 518060, China; cliao@szu.edu.cn

**Keywords:** anticancer peptides, sequence-based method, g-gap dipeptide, 400D, dimension reduction

## Abstract

Cancer is a serious health issue worldwide. Traditional treatment methods focus on killing cancer cells by using anticancer drugs or radiation therapy, but the cost of these methods is quite high, and in addition there are side effects. With the discovery of anticancer peptides, great progress has been made in cancer treatment. For the purpose of prompting the application of anticancer peptides in cancer treatment, it is necessary to use computational methods to identify anticancer peptides (ACPs). In this paper, we propose a sequence-based model for identifying ACPs (SAP). In our proposed SAP, the peptide is represented by 400D features or 400D features with g-gap dipeptide features, and then the unrelated features are pruned using the maximum relevance-maximum distance method. The experimental results demonstrate that our model performs better than some existing methods. Furthermore, our model has also been extended to other classifiers, and the performance is stable compared with some state-of-the-art works.

## 1. Introduction

Cancer is a serious health issue worldwide [1,2], and millions of people die of it. Traditional treatment methods focus on killing cancer cells, but at the same time normal cells are also killed and there are high costs involved [3,4]. However, this situation has changed with the discovery of anticancer peptides (ACPs). Because ACPs can interact with the anionic cell membrane components of cancer cells, cancer cells can be killed selectively by the ACPs without impairing the normal cells [5,6]. Anticancer peptides do not impair the body’s physiological functions, providing a new direction for cancer treatment. Though there exist drawbacks in the development process of ACPs, ACPs are safer than synthetic drugs, and have greater efficacy, selectivity, and specificity [7,8]. ACPs represent a promising line of treatment [5,6]. Thus, treatment methods involving anticancer peptides have been receiving increasing attention. ACPs are represented by short peptides with 5 to 30 amino acids. However, it is still difficult to distinguish ACPs from other (natural or artificially designed) peptides. It is quite expensive and time-consuming to identify anticancer peptides using experimental methods. Moreover, only few of them can be applied in clinics [9]. Therefore, it is necessary to use computational methods to predict anticancer peptides. The identification of ACPs could prompt their application in cancer treatment, so it is urgent to use computational methods to predict anticancer peptides.

There have been some works on identifying ACPs by computational methods. Tyagi et al. [10] used the support vector machine for classifying the type of ACPs, in which amino acid composition and binary profiles are considered as features to represent the peptides. Hajisharifi et al. [11] proposed a model based on the local alignment kernel method to predict the ACPs. Chen et al. [12] developed a powerful sequence-based method to discriminate anticancer ACPs, and better results were demonstrated through cross validation. All the mentioned methods have reported encouraging results for ACP prediction. 

However, for the purposes of prompting the application of ACPs into cancer treatment quickly, it is important to develop an efficient model for predicting ACPs. The experimental results show that sequence-based methods [13,14,15,16] perform better than the previous methods [10,11] by considering the sequence pattern information. In our work, the peptides are represented by Pseudo amino acid composition (PseACC) g-gap dipeptide mode and 400D features. The g-gap dipeptide model [12] is a sequence-based feature which describes the occurrence frequency of the g-gap dipeptide. Meanwhile, 400D is a feature of the occurrence frequency of consecutive amino acid residue in the proteins. Afterwards, the features are reduced by using the maximum relevance-maximum distance method [17]. Then, the unrelated features are pruned by maximum distance. Moreover, the model is applied to three classifiers, such as random forest, ensemble classification, and support vector machine, respectively. The model performs more stably than some existing methods with respect to some performance metrics. 

This work provides several main contributions. Firstly, we proposed a sequence-based model (called sequence-based model for identifying ACP (SAP)) for predicting ACPs, which performs stably on different classifiers. Secondly, in our proposed SAP, the peptides are represented by PseACC with g-gap dipeptide composition mode and 400D features, which can describe the sequence pattern information. Thirdly, features are pruned by the maximum relevance-maximum distance method without affecting the performance of the predictor. 

Section 2 introduces the data sets used for the experiments and the methods for identifying ACPs. The results are compared in Section 3. Finally, our work is concluded in Section 4 and Section 5.

## 2. Material and Methods

### 2.1. Integration of Anticancer and Non-Anticancer Peptide Sources

In this section, the construction of the benchmark data set is introduced. Though there are larger data sets [18], to compare with related work, we used the data set in [19]. The data set includes anticancer peptides and non-anticancer peptides. The set of anticancer peptides is represented as C^+^, and the non-anticancer peptides are represented as C^−^. Thus, the union of the C^+^ and C^−^ is the benchmark data set.

(1) $$C=C^{+}\cup C^{-}$$

According to the property of data sets, the intersection of the C^+^ and C^−^ should be empty. Hence, there is

(2) $$\Phi =C^{+}\cap C^{-}$$

Set C^+^ contains 138 anticancer peptides, which are selected from the data set of Chen et al. [19]. The 138 positive samples are selected from the antimicrobial peptides database [20]. To reduce redundancy and avoid homology bias, peptides with more than 90% sequence similarity to each other are removed from the data set by Cluster Database at High Identity with Tolerance (CD-HIT) [21]. CD-HIT is a type of software used for reducing the similarity of the protein sequences. Finally, there are 206 non-anticancer peptides and 138 anticancer peptides in data set C for the experiments.

### 2.2. Features for Peptide Representation

**Figure 1:**
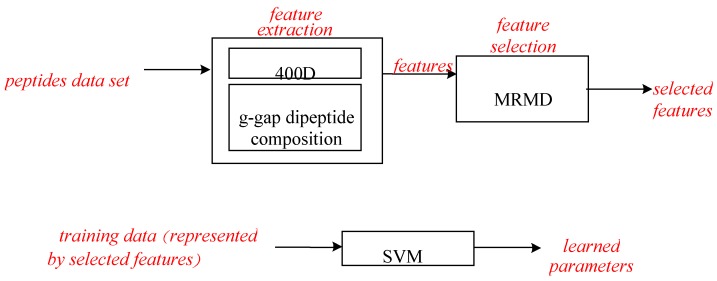
The flow chart of identifying anticancer peptides. MRMD: maximum relevance-maximum distance; SVM: support vector machine.

The features used for peptides representation are introduced in this section. The flow chart of our sequence-based identifying anticancer peptides model is illustrated first. Figure 1 shows the peptides are represented by the features (i.e., 400D or g-gap dipeptide composition). Then, the features are selected by the maximum relevance-maximum distance (MRMD) method. It denotes the maximum relevance-maximum distance method for dimension reduction. The features are ranked by the MRMD method, and then some features are pruned for the purpose of balancing the accuracy and stability of feature ranking and the prediction task. The MRMD method will be illustrated in detail in Section 2.3 (Feature Selection). The peptides are predicted by the classifiers (i.e., the support vector machine (SVM) in our model).

In our SAP, the sequence pattern information is represented by 400D features and PseACC with g-gap dipeptide composition. The 400D features will be introduced first. The features of PseACC with g-gap dipeptide composition will be described later. 

There is a peptide sample P with L residues, and a straightforward method for peptide P representation is

P = R_1_R_2_R_3_…R_L_ (3)

where R1 is the 1-st residue of P, and RL is the l-th residue of the peptide. fi is used to represent the normalized occurrence frequency of the i-th type of native amino acid in the peptide. Thus, the peptide P is represented by R = [f_1_,…f_i_,…,f_20_]. However, the sequence information is lost in the frequency feature. 

In contrast to previous works, the proposed model considers the sequence information (Tyagi et al., 2014). The 400D features are sequence-based features. There are 20 amino acids used to represent the protein, so the combination of two consecutive amino acids is represented as AB. The frequency of the combination of AB is denoted as f_AB_. Thus, there are 400(20^2^) possible combinations of each two amino acids. The 400D features are represented by the frequencies of the 400 combinations. Thus, the value of each dimension of 400D represents the occurrence times of each two consecutive amino acids. The pattern information is described in the 400D features. 

Pseudo amino acid composition [22,23] and Chou’s PseACC [24,25,26] are usually used to extract the sequence pattern information of the protein. Some more recent special protein identification methods [27,28,29] also use the features. Pseudo amino acid composition [22] has been used in many fields of protein attribute predictions [27,28,30,31,32,33,34,35,36,37,38,39,40,41,42], as well as in drug development [43] and studies on the drug target area [44,45]. In contrast to previous works, g-gap dipeptide composition is considered in Chen’s work [12]. Given the peptide P as shown in Equation (3), the g-gap dipeptide composition is shown as

(4) $$P=\left[d_{1}^{g}d_{2}^{g}\ldots \;d_{u\;}^{g}\ldots d_{u}^{400}\right]$$

where $d_{u}^{g}$ denotes the occurrence frequency of the u-th g-gap dipeptide in P. $d_{u}^{g}$ is calculated by Equation (5) [12].

$$P=\left[d_{1}^{g}d_{2}^{g}\ldots \;d_{u\;}^{g}\ldots d_{u}^{400}\right]=\;\{\begin{array}{l} proximate\;dipeptide\;composition,\;g=0\\ one-gap\;dipeptide\;composition,\;g=1\\ two-gap\;dipeptide\;composition\;g=2\\ three-gap\;depeptide\;composition,\;g=3\\ four-gap\;dipeptide\;composition,\;g=4 \end{array}$$

(5) $$d_{u}^{g}=\frac{n_{u}^{g}}{\sum_{u=1}^{400}n_{u}^{g}}=\frac{n_{u}^{g}}{L-g-1}$$

where $n_{u}^{g}$ is the number of the u-th g-gap dipeptide. For the short peptides, the range of g is usually up to 4. When g equals 0, the dipeptide composition is formed by the nearest residues. When g is 1, the second nearest residues are considered, and so forth. 

The features of 400D and pseudo amino acids are integrated together to represent the peptide P. A redundancy may exist between the features, and thus the unrelated features are pruned by the MRMD method [17]. 

### 2.3. Feature Selection

The objective function of MRMD is shown as Equation (6). If m-1 features have been selected, the m-th feature will be selected if the i-th feature maximizes Equation (6).

(6) $$\max\left(MR_{i}+MD_{i}\right)$$

where MR_i_ is the relevance between the features. The relevance is measured by the Pearson’s correlation coefficient, shown as Equation (7).

(7) $$PCC\left(\vec{X},\vec{Y}\right)=\frac{\sum_{k=1}^{N}\left(x_{k}-\bar{x}\right)\left(y_{k}-\bar{y}\right)}{\sqrt{\sum_{k=1}^{N}\left(x_{k}-\bar{x}\right)}\sqrt{\sum_{k=1}^{N}\left(y_{k}-\bar{y}\right)}}$$

where N is the number of vectors, and $\bar{x}\left(\bar{y}\right)$ is the average value on the k-th dimension. MD_i_ is used to measure the level of similarity between two feature vectors. In our experiments, the maximum distance is calculated as the mean of the Euclidean distance (ED), cosine distance (COS) and Tanimoto coefficient (TC) (shown as Equation (11)). The maximum distances used are defined as follows.

(8) $$ED_{i}=\frac{\sum ED\left({\vec{F}}_{i},{\vec{F}}_{k}\right)}{M-1}=\frac{\sum \sqrt{\sum_{k=1}^{K}{\left(x_{i}-x_{k}\right)}^{2}}}{M-1}\;\left(1\leq k\leq M,k\neq i\right)$$

(9) $$\;COS_{i}=\frac{\sum cos\left({\vec{F}}_{i},{\vec{F}}_{k}\right)}{M-1}=\frac{\sum {\vec{F}}_{i}{\vec{F}}_{k}/\left|\left|{\vec{F}}_{i}\right|\right|\left|\left|{\vec{F}}_{k}\right|\right|}{M-1}\;\left(1\leq k\leq M,k\neq i\right)$$

(10) $$TC_{i}=\frac{\sum TC\left({\vec{F}}_{i},{\vec{F}}_{k}\right)}{M-1}=\frac{\sum {\vec{F}}_{i}{\vec{F}}_{k}/({\left|\left|{\vec{F}}_{i}\right|\right|}^{2}+{\left|\left|{\vec{F}}_{k}\right|\right|}^{2}-{\vec{F}}_{i}{\vec{F}}_{k})}{M-1}\;\left(1\leq k\leq M,k\neq i\right)$$

(11) $$maxMD_{i}=\frac{1}{3}\left(ED_{i}+COS_{i}+TC_{i}\right)\;\left(1\leq i\leq M\right)$$

In Equations (8)–(11), M is the number of features. The distance on each dimension is calculated, and the feature with the maximum distance will be selected by satisfying Equation (6).

### 2.4. Classification Methods

The basic idea of classification is learning the parameters of the classifier by the training data. There are N tuples in the training set $\;TS=\left\{\left(s_{1},y_{1}\right),\ldots ,\left(s_{N},y_{c}\right)\right\}$, and $\left(y_{1},\ldots y_{c}\right)\in C$. C is the label set of the data. C is the number of classes. Each sample $s_{i}\left(1\leq i\leq N\right)$ is represented by a multi-dimensional vector, such as $s_{i}=\left(d_{1},\ldots ,d_{M}\right)$, where M is denoted as the dimension number. The goal of classification is to train the parameters of the classifier by the training set with the minimum accuracy loss.

In our SAP, the SVM is used to classify the anticancer peptides and non-anticancer peptides. The objective function of SVM is shown as Equation (12) [46]. The goal of SVM is to find a hyperplane (w) that can maximize the distance between the samples of different classes.

(12) $$max\frac{1}{\left|\left|w\right|\right|}$$

$$s.t.\;y_{i}\left(w^{T}s_{i}+b\right)\geq 1,i=1,\ldots ,N$$

where $y_{i}$ is the label of the training sample $s_{i}$, and b is the bias.

### 2.5. Evaluation Criteria and Measurement

The evaluation criteria are introduced in this part. Five metrics are used to evaluate the performance of the predictors, which are specificity (SP), sensitivity (SN), overall accuracy (Acc), Mathews correlation coefficient (MCC), and the *F*-score, respectively. $N^{+}$ is denoted as the number of anticancer peptides labeled by the classifier, and $F^{-}$ is the number of anticancer peptides misclassified by the non-anticancer peptides. $N^{-}$ is denoted as the number of non-anticancer peptides labeled by the classifier, and $F^{+}$ is the number of non-anticancer peptides labeled by the anticancer peptides.

Sensitivity is used in Chou’s work [47] and represents the sensitivity, which is calculated by Equation (13). Specificity is the specificity of the algorithm, which is measured by the rate of misclassification of the anticancer peptide. The calculation of Sp is shown as Equation (14). Assessments of Sp or Sn individually are not sufficient to evaluate the performance of a method. The overall accuracy is calculated by Equation (15). Mathews correlation coefficient considers the rate of both Sp and Sn, as shown in Equation (16).

(13) $$Sn=1-\frac{F^{-}}{N^{+}}$$

(14) $$Sp=1-\frac{F^{+}}{N^{-}}$$

(15) $$Acc=1-\frac{F^{+}+F^{-}}{N^{+}+N^{-}}$$

(16) $$MCC=\frac{1-\left(\frac{F^{-}}{N^{+}}+\frac{F^{+}}{N^{-}}\right)}{\sqrt{\left(1+\frac{F^{+}-F^{-}}{N^{+}}\right)\left(1+\frac{F^{-}-F^{+}}{N^{-}}\right)}}$$

There are *u* peptides labeled by anticancer peptides, and there are *v* real anticancer peptides in u. Precision (P) is P=vu. There are *v* real anticancer peptides labeled by the classifier, and there are *w* anticancer peptides in the data set. The recall (R) is R=vw. Precision and recall are considered in *F*-score [48]. The calculation of the *F*-score is shown in Equation (17).

(17) $$F-Score=\frac{2*P*R}{P+R}$$

The performance of the methods is measured by the abovementioned five metrics. Accuracy is the average accuracy of the method. Mathew’s correlation coefficient describes the stability of the algorithms. *F*-score reflects the trade-off between the precision and accuracy.

## 3. Results

### 3.1. Contrast Experiments Based on 400-Dimensional Classical Features

Experiment (1): The experiments are running on iACP (ACP identifying tool) and SAP. The results are reported based on ten cross validations. Table 1 shows the classification performance of our model compared with iACP [19].

The experimental results show that our proposed method by using 400D features performs better than iACP [19] on all the five metrics. The MCC value for our method is 0.8301, and the MCC value of iACP is 0.8058. iACP is a predictor based on the SVM, and the peptide is represented by g-gap dipeptide model. Our model improves the MCC of iACP by nearly 3%. The *F*-score of SAP is 0.8947 while the *F*-score of iACP is 0.8788. The *F*-score of iACP is improved by 1.6% using our method. The experimental results show that our method can identify the anticancer peptides accurately. 

### 3.2. Contrast Experiments Based on Integrated Features

Experiment (2): The experiments are run on the selected integrated features of the data set. The peptides are represented by the 400D and g-gap dipeptide features used in [19]. Each peptide is described by a high-dimensional vector. The features will be pruned by the maximum relevance-maximum distance methods. Table 2 shows the results of the experiments.

The results of Experiment (2) show that the proposed SAP still performs the best compared with two other algorithms. However, the accuracy of the selected SAP, whose features are pruned by the maximum relevance-maximum distance method, is comparable to that of iACP, which means that the features of selected SAP can also classify the peptides well. The specificity of the selected SAP reaches to 0.966, which is better than for SAP and iACP.

### 3.3. Comparison with State-of-the-Art Methods

**Figure 2:**
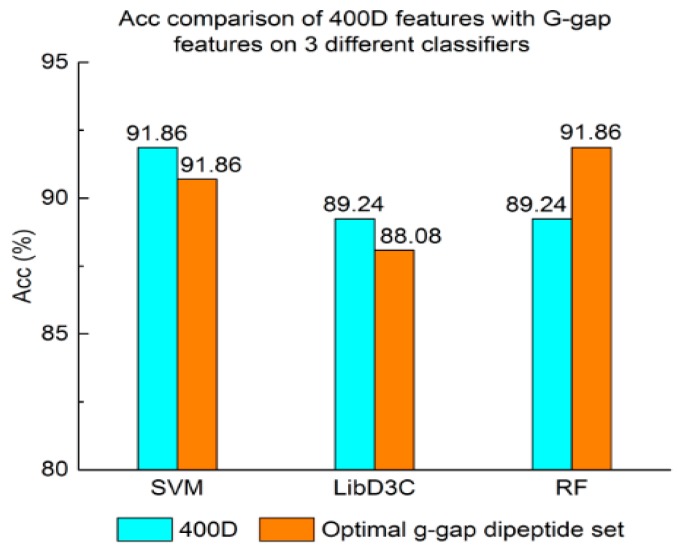
Overall accuracy comparison of 400D features with G-gap features on three different classifiers. RF: random forest.

**Figure 3:**
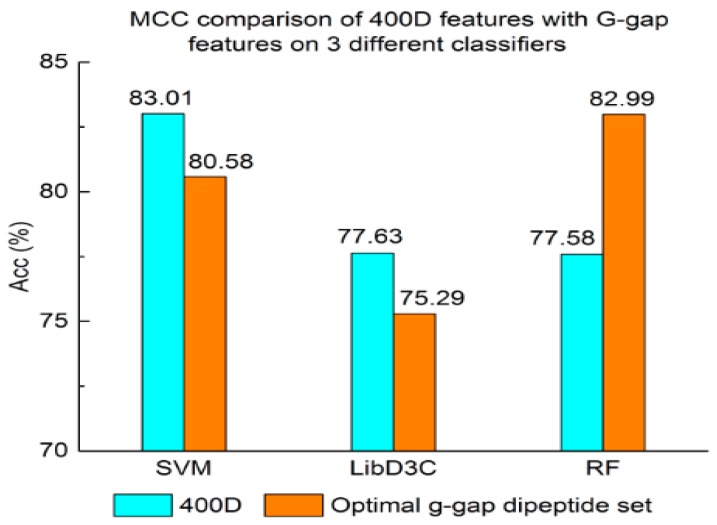
Mathew’s correlation coefficient value comparison of 400D features with G-gap features on three different classifiers.

**Figure 4:**
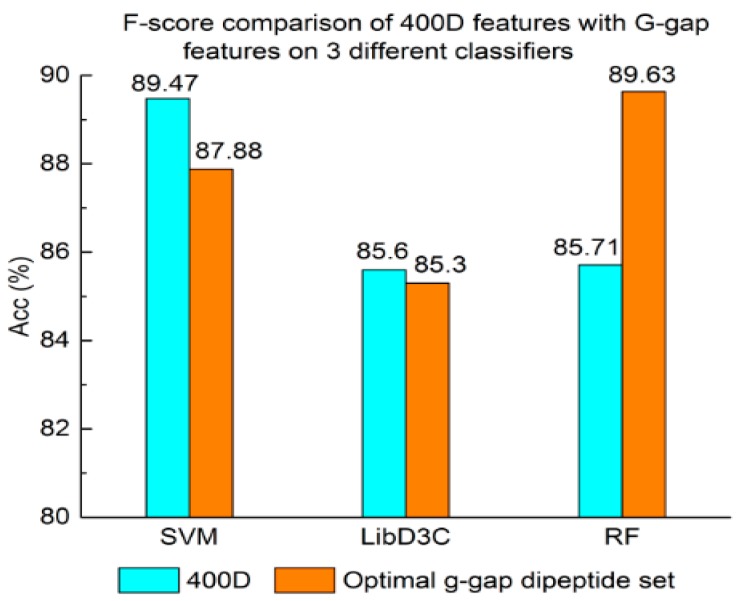
*F*-score comparison of 400D features with G-gap features on three different classifiers.

Experiment (3): For the purpose of demonstrating the efficiency of SAP used in our method, the proposed model is compared with the features used in iACP [19]. Figure 2, Figure 3 and Figure 4 show the metrics of the Acc, MCC, and *F*-score of 400D features compared with g-gap dipeptide composition on three classifiers (support vector machine, random forests, and LibD3C [49]).

The random forest (RF) ensemble algorithm [50,51] trains a few decision trees together. The training samples are selected by bagging sampling, which means that the samples will be put back into the data set after each selection. The training samples on each decision tree can be overlapped. The key idea of random forest is m features are selected from M dimensions on each decision tree, and t decision tree will be trained. Then, a decision is made by a voting process. 

In random forest, the features are evaluated by the information gain. The information gain is used to find the features and the threshold, and the formula is shown in Equation (18) [50,51].

(18) $$I\left(s_{i}=N^{+}\right)=-log_{2}P\left(N^{+}\right)$$

where s_i_ is the i-th sample in the training set, and $P\left(N^{+}\right)$ is the probability of class $N^{+}$.

LibD3C is a selective ensemble model. In the model, a number of candidate classifiers are trained, and the classifiers which are accurate and diverse will be selected. LibD3C is a hybrid model of ensemble pruning which is based on *K*-means clustering and the combination of dynamic selection and circulating [49].

**Figure 5:**
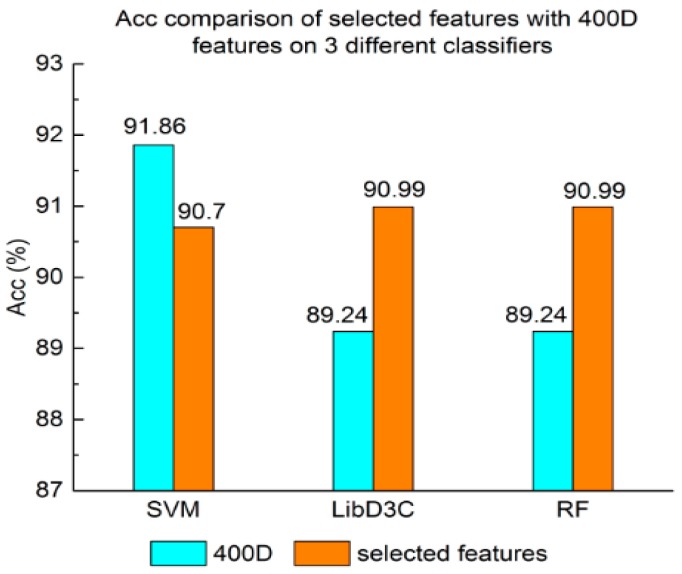
Acc comparison of selected features with 400D features on three different classifiers.

**Figure 6:**
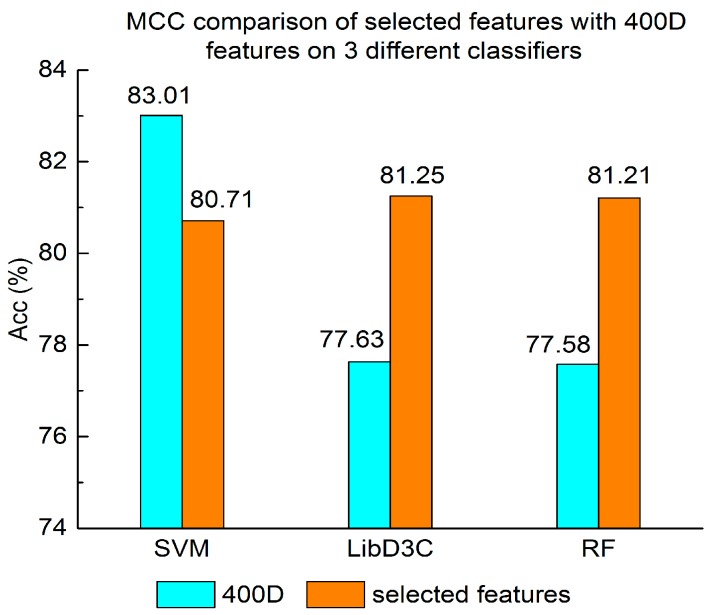
MCC comparison of selected features with 400D features on three different classifiers.

**Figure 7:**
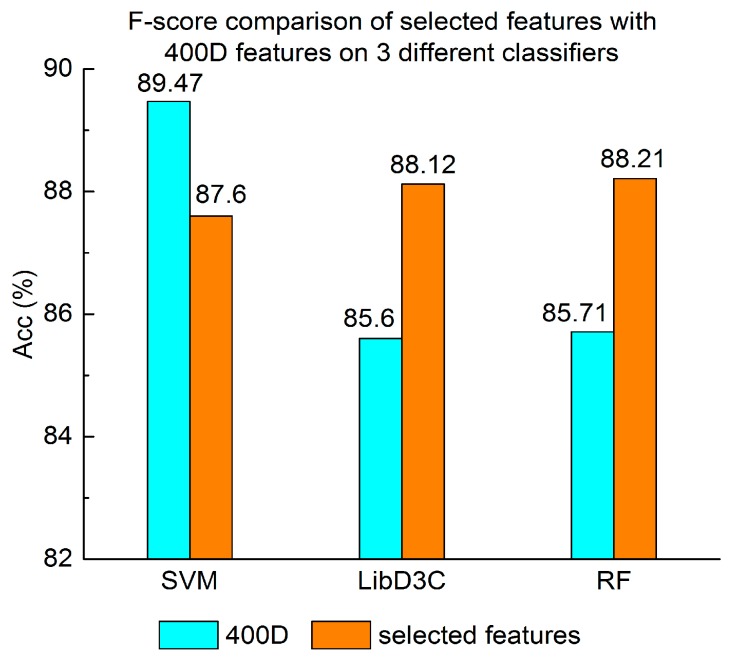
*F*-score comparison of selected features with 400D features on three different classifiers.

The performance of 400D compared with g-gap features is shown in Figure 2, Figure 3 and Figure 4. The 400D features perform better when SVM or LibD3C is used. However, on the RF algorithm, the method of 400D features does not perform better than g-gap features on Acc, MCC, and *F*-score. However, in the experiments the performance of RF using the selected features is compatible to that of RF using g-gap features (shown in Figure 5, Figure 6 and Figure 7). In the experiments, the g-gap features perform best on the RF classifier. The accuracy of 400D using SVM and g-gap with the RF algorithm is the best, at 0.9186. The lowest accuracy is g-gap on the LibD3C. The best MCC is 0.8301, which uses 400D features on the SVM. The lowest MCC is 0.7529 when g-gap features are used to classify the peptides by LibD3C. The lowest *F*-score is 0.853 when LibD3C is used with g-gap features. The highest *F*-score is 0.8963 when RF classifies the peptides with g-gap features.

Experiment (4): The performance of selected integrated features compared with 400D features is shown in Figure 5, Figure 6 and Figure 7. The 400D features perform better when SVM is used. However, on the LibD3C and RF, the selected features perform better than 400D features on Acc, MCC, and *F*-score. However, when the ensemble algorithms are used, the methods with selected features perform better than the 400D features on Acc, MCC and *F*-score. 

Above all, the experimental results show the method of selected features performs better on ensemble classifiers (RF and LibD3C) than 400D features, and 400D features perform better than g-gap features on SVM and LibD3C.

## 4. Discussion

We compared the results of Experiments (1–4). First, the 400D SVM performs better than iACP, as shown in Table 1. Both the 400D features and g-gap features can represent the sequence pattern information of peptides, but 400D can classify the peptides more accurately. Thus, we propose a new method for peptide classification.

Second, the method based on 400D features performs stably on three different classifiers. The method based on 400D features is flexible on the classifiers. By comparing the experimental results of the two groups, we draw the conclusion that the 400D features can represent the sequence information of the anticancer peptide. The method with selected features can improve the performance of the method based on 400D features on RF.

Since user-friendly and publicly accessible web servers represent future directions for developing practically more useful models, we shall make efforts in our future work to provide a web server for the method presented in this paper. Moreover, as demonstrated in a series of recent publications (see, e.g., [1,52,53,54,55,56]) on the development of new prediction methods, user-friendly, and publicly accessible web servers will significantly enhance their impacts, and we shall make efforts in our future work to provide a web server for the prediction method presented in this paper.

## 5. Conclusions

In this paper, a novel hybrid sequence-based model for identifying anticancer peptide prediction is proposed. In our proposed model, 400D is used to represent the sequence pattern information. In contrast to previous works, the redundancy features and unrelated features are pruned. In the experiments, the model based on 400D features performs better than existing methods. The experimental results demonstrated that the 400D model performs stably on the three classifiers, because the poorest performance is shown when g-gap is used. The features selected by the MRMD method can improve the performance of 400D features on RF. Our proposed method shows better performance with respect to anticancer peptide classification. On the other hand, there are also some related problems that our method can be used to address, such as DNA-binding protein prediction [52], methylation site prediction [57], phosphorylation site prediction [58] and protein–protein interaction prediction [59], etc.

## Figures and Tables

**Table 1:** Performance comparison with state-of-the-art methods.

Methods	Sn	Sp	Acc	MCC	*F*_score
iACP	84.06%	95.15%	90.7%	80.58%	87.88%
SAP (400D)	86.23%	95.63%	91.86%	83.01%	89.47%

Sn: sensitivity; Sp: specificity; Acc: overall accuracy; MCC: Mathew’s correlation coefficient; SAP: sequence-based model for identifying ACP; iAPC: tool for identifying ACP proposed in [19].

**Table 2:** Performance comparison with selected features.

Methods	Sn	Sp	Acc	MCC	*F*_score
iACP (g-gap)	84.06%	95.15%	90.7%	80.58%	87.88%
SAP (400D)	86.23%	95.63%	91.86%	83.01%	89.47%
SAP (selected features)	81.88%	96.6%	90.7%	80.71%	87.6%
